# Distinct Difference in the Geometries of NCCL^–^ Anions (L = N_2_, CO, CS): A Balance Between π Conjugation and Steric Repulsion

**DOI:** 10.1021/acs.inorgchem.5c04439

**Published:** 2025-10-29

**Authors:** Jia Wei, Rui Ma, Jinshuai Song, Yandong Duan, Xiaoyan Li, Huaiyu Zhang, Yirong Mo

**Affiliations:** † Institute of Computational Quantum Chemistry, and Hebei Key Laboratory of Inorganic Nanomaterials, College of Chemistry and Materials Science, 66447 Hebei Normal University, Shijiazhuang 050024, China; ‡ Green Catalysis Center, and College of Chemistry, 12636 Zhengzhou University, Zhengzhou 450001, China; § Hebei Key Laboratory of Photoelectric Control on Surface and Interface, School of Sciences, 83524 Hebei University of Science and Technology, Shijiazhuang 050018, China; ∥ Department of Nanoscience, Joint School of Nanoscience and Nanoengineering, 14616 University of North Carolina at Greensboro, Greensboro, North Carolina 27401, United States

## Abstract

NCCL^–^ anions (L = N_2_, CO and CS) exhibit notable geometric differences: NCCNN^–^ favors a bent structure, whereas NCCCO^–^ and NCCCS^–^ prefer linear configurations. Here, we investigate the origin of their geometric differences using the conventional density functional theory (DFT) and block-localized wave function (BLW) method. Computations reveal that the bent structure is preferred for all species at the BLW state in which the in-plane π_//_ back-donation from NCC^–^ to ligands is disabled. For NCCNN^–^, enforcing linearity leads to a gain in the π_//_ electron delocalization (conjugation) stability, but it is insufficient to offset the steric penalty. Differently, NCCCO^–^ and NCCCS^–^ experience reduced steric repulsion in linear geometries, thereby favoring more linear geometries as their energy minima. “*In situ*” orbital correlation diagrams reveal an orbital swap in NCC^–^ with the approaching of the ligand L and confirm the carbone theory proposed by the Frenking group. Overall, the geometries (or the degree of bending) of NCCL^–^ anions are governed by the balance between the π_//_ conjugation and steric repulsion. Substituting the electron-withdrawing NC group with CH_3_ or F, which provides in-plane σ-electrons or lone pairs, further bends the ∠RCL angle in RCNN^–^ anions, while RCCO^–^ and RCCS^–^ retain their nearly linear forms.

## Graphical abstract

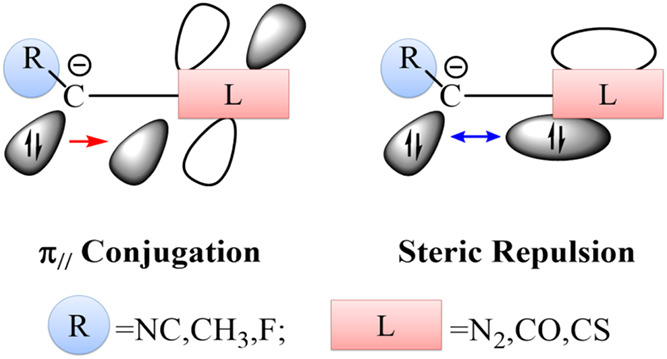

## Introduction

Electron delocalization (also known as resonance or electron transfer), a fundamental concept in chemistry, occupies a central place in both chemical theory and education. Based on the definition provided by IUPAC in 1994, electron delocalization is a quantum mechanical phenomenon in which electrons are not confined to individual bonds or atoms but extend across the molecular framework.^1^ This spatial spreading of electron density confers significant energetic stabilization (e.g., resonance energy) and profoundly influences key properties of molecules and materials such as electronic conductivity, optical absorption and emission profiles, charge transport, magnetic behavior, and chemical reactivity. Understanding the nature and extent of electron delocalization is thus critical for rationalizing chemical and physical properties and for the rational design of novel molecules and materials. Computational chemistry provides powerful analytical tools to dissect and quantify electron delocalization. For instance, natural bond orbital (NBO)^2^ and adaptive natural density partitioning (AdNDP)^3^ methods provide orbital-based analyses that visually demonstrate the characteristics of delocalization. Electron density topology approaches, notably quantum theory of atoms in molecules (QTAIM),^4,5^ quantify electron sharing via delocalization indices and bond critical point analysis. Specialized multicenter indices such as para-delocalization index (PDI)^6^ and aromatic fluctuation index (FLU)^7^ directly measure spatial electron distribution, while the electron localization function (ELF)^8,9^ visualizes localized versus delocalized domains. Magnetic criteria, primarily nucleus-independent chemical shift (NICS)^10,11^ calculations, probe ring-current effects to evaluate aromatic delocalization. From an energetic perspective, NBO quantifies delocalization through second-order perturbative estimates of donor–acceptor interactions,^12^ while *ab initio* valence bond (VB) methods^13,14^ can explicitly compute resonance energies by enforcing electron localization.

In this work, we focus on ketenyl anions/ynolates RCCO^–^, which are conjugated systems in which π electron delocalization plays an important role in their structures and properties. The development of ketenyl anions/ynolates RCCO^–^ offers an alternative and potentially more versatile route to ketenes, as RCCO^–^ anions are manageable precursors that can be transformed into ketene derivatives under milder settings.^15^ Posphinoyl-substituted,^16–18^ phosphino-substituted,^19^ tosyl-substituted,^20^ and cyano-substituted^21^ ketenyl anions have been isolated by a mild ligand-exchange reaction at the carbon atom. Most recently, Gessner and Frenking *et al*.^22^ isolated a few valence-isoelectronic compounds of NCCCO^–^, including the cyanodiazomethanide anion (NCCNN^–^) and cyanothioketenyl anion (NCCCS^–^) (see Scheme 1). Notably, the NCCNN^–^ anion exhibits a distinct bent structure with a ∠CCN angle of experimentally 133° (in crystal) and computationally 120° (CCSD­(T)) or 128° (BP86), while both experimental and computational studies revealed a flatter geometry around the central carbon atom for NCCCO^–^, featuring a ∠CCC angle of 166° (exptl) and 151° (CCSD­(T)) or 180° (BP86), respectively.^21,22^ Both CCSD­(T) and BP86 calculations suggest that NCCCS^–^ adopts a linear structure. Similar to carbones CL_2_ and their isoelectronic homologues,^23–25^ the electronic structure of NCCL^–^ (ligand L = N_2_, CO, CS) can be interpreted in terms of NCC^–^←L σ-donation and in-plane and out-of-plane NCC^–^ → L π-back-donation interactions.^22^ Further, the varying bent angles observed in these species are correlated with the magnitude of the π-back-donation from carbon to ligand, which increases in the order N_2_ < CO < CS.^22^ We note that other isoelectronic compounds related to NCCCO^–^, such as OCNCO^+^,^26^ OCCCO,^27–29^ and OCBCO^–^,^30^ have also been extensively investigated. It has been revealed that the degree of bending in these species increases from the central atom N to C to B due to the progressive π-back-donation capability of the central atom.^30^ In addition, phosphinoyl-substituted ketenyl anions exhibit flatter geometries, with the ∠PCN angles ranging from 142.6 to 160.1°,^17^ which are notably larger than the ∠PCC angles found in diazomethanide analogues.^31^ This parallels the structural trend observed in the NCCL^–^ anions.

**Scheme 1:**
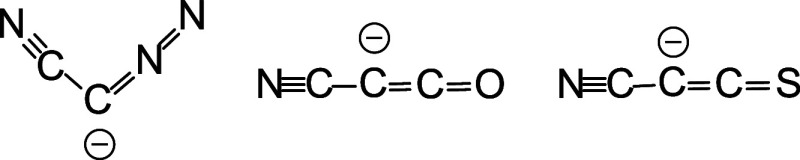
Cyanodiazomethanide Anion (NCCNN^–^), Cyanoketenate­(NCCCO^–^), and Cyanothioketenyl Anion (NCCCS^–^)

However, there is a duality for π electrons.^32^ While electron delocalization is well-recognized as a stabilizing force, there is simultaneous electron repulsion, which is destabilizing and can profoundly impact the geometrical and electronic structures of conjugated compounds by offsetting the delocalization stability. This repulsion, arising from the Pauli exclusion principle (preventing electron orbital overlap) and electrostatic repulsion and thus summarily the steric repulsion, establishes critical spatial constraints. To highlight this specific repulsion among π bonds, in 2016, we coined the new concept of intramolecular multibond strain for cumulenes and beyond.^33^ Subsequent studies revealed that the significant π–π repulsion influences the C–N and N–N bond length in nitrobenzene and N_2_O_4_.^34^ Similarly, in dialumenes and disilenes bearing two amino groups, the stretched and/or extremely trans-bent Si=Si or Al=Al bonds, compared to aryl- or silyl-substituted species, primarily originate from the repulsion between the lone pairs on nitrogen and the Si=Si or Al=Al π bond, with conjugation contributing modestly to bond lengthening.^35^ Additionally, Schoeller highlighted the repulsion between the lone pairs at the amino groups and the central π bond to explain the preference of a bisorthogonal over a coplanar conformation in diphosphene, disilene, and diimine.^36^

Motivated by the above works, we are interested in whether the geometries of NCCL^–^ anions (L = N_2_, CO and CS) are governed by the repulsion between the ligand’s in-plane π-bond and the electrons around the central carbon atom, such as the σ_C–C_ bond, localized in-plane lone pair or π_C–C_ bond, or alternatively, by the in-plane π-back-donation from the central carbon to the ligand. To address this question and offer new insights into the structural and electronic properties of these species, we employed an *ab initio* VB^13,14^ approach, or the block-localized wave function (BLW) method^37–40^ at the DFT level, complemented by the natural steric analysis (NSA).^41,42^ Furthermore, to probe the substituent effect, we extended our study from NCCL^–^ to RCL^–^ anions by replacing the electron-withdrawing NC group with other substituent groups CH_3_ or F, which contribute in-plane σ-electron or lone pairs, respectively.

## Theoretical Methods and Computational Details

The applications of quantum mechanics to molecular systems lead to two general theories, including molecular orbital (MO) theory and VB theory. They differ primarily in the use of one-electron orbitals, as MO theory adopts delocalized orthonormal orbitals while VB theory uses localized nonorthogonal orbitals.^13,14^ One of the significant merits of VB theory is its lucid pictures for chemical bonding, but MO theory enjoys efficient implementations. To combine the advantages of both MO and VB theories, we developed the BLW method.^37–39^ Within the BLW method, all electrons and primitive basis functions are partitioned to several subgroups (blocks), and all orbitals are expanded in only one block and thus, block-localized. The orbitals in the same block are constrained to be orthogonal, just as in MO theory, but orbitals belonging to different blocks are nonorthogonal, as in VB theory. The BLW method is the simplest variant of *ab initio* VB theory and is available at the DFT level. This enables strict localization of electronic states, thereby permitting a rigorous assessment of electron delocalization effects on molecular stability and geometry.^37–39,43^

The energy decomposition (BLW-ED) approach based on the BLW method can decompose the binding energy into a few physically meaningful components. In this BLW-ED approach,^44,45^ the intermolecular binding energy among RC^–^ and L is composed of two parts, namely, the deformation energy (Δ*E*_def_) from their respective free and optimized monomer structures to the distorted geometries in the optimal complex structure and the interaction energy (Δ*E*_int_) among monomers as

1 $$\Delta E_{b}=\Delta E_{\mathrm{def}}+\Delta E_{\mathrm{int}}$$

The latter can be further decomposed into three terms as

2 $$\Delta E_{\mathrm{int}}=\Delta E_{\mathrm{steric}}+\Delta E_{\mathrm{pol}}+\Delta E_{\mathrm{CT}}$$

where Δ*E*_steric_ is a combination of electrostatic, Pauli exchange interactions, and the electron correlation included in DFT; Δ*E*_pol_ is the stabilizing polarization energy corresponding to the redistribution of electron densities within individual monomers due to the electric fields imposed by others; and Δ*E*_CT_ is the charge transfer stabilization energy resulting from the penetration of electrons among monomers after the basis set superposition error (BSSE) correction.^46^

In accordance with a well-established physical picture of “steric repulsions”, NSA expresses steric exchange repulsion as the energy difference due to orbital orthogonalization.^41,42^ In this work, the NSA was used to check the repulsion between the ligand’s in-plane π-bond and the electrons around the central carbon atom, such as the σ_C–C_ bond, in-plane localized lone pair, or π_C–C_ bond.

All regular DFT and subsequent BLW calculations for naked anions at the M06-2X-D3/6-311+G­(d)^47^ level and the CASPT2 calculations for NCC^–^ were performed with the GAMESS^48,49^ software to which the BLW code has been ported in our laboratories. NBO analyses including NSA were conducted with the NBO 6 program.^50^ Gaussian16 software^51^ was used for the CCSD­(T) calculations for naked anions and the DFT calculations in the presence of counter cations. For fully optimized geometries, harmonic vibrational calculations were performed in order to assess the nature of the stationary points on the potential energy surfaces. The energy profiles along the angle ∠RCL were evaluated by performing constrained geometry optimizations at the M06-2X-D3/6-311+G­(d) level, in which only the bond angle was fixed at a series of values, with all of the rest of the geometrical parameters optimized.

## Results and Discussion

### Geometries and Electronic Structures of NCCL^–^ Anions

The optimized structures of NCCL^–^ (L = N_2_, CO, and CS) anions are shown in Figure 1. The bond angles ∠RCL in NCCL^–^ (L = N_2_, CO, and CS) anions (120.6, 179.5, and 177.3°, respectively) are consistent with previously reported values (128.4, 180.0, and 180.0°) at the BP86+D3­(BJ) level.^22^ For NCCNN^–^, the ∠RCL bond angle computed at the M06-2X-D3 level also agrees well with the CCSD­(T) result. But for NCCCO^–^ and NCCCS^–^, the ∠RCL angles at the DFT level are slightly larger than the values obtained at the CCSD­(T) level. This is because the molecular potential energy surfaces associated with the ∠RCL angle are very flat or sensitive to electron correlations. Constraining ∠RCL to linearity costs only 1.2 and 0.4 kcal/mol for L = CO and CS, respectively, with negligible effects on C_1_–C_2_ and C_1_–C_3_ bond lengths (see Table S1) at the CCSD­(T) level. In addition, all isolated ketenyl anions reported to date feature lithium, sodium, or potassium counterions. To evaluate the influence of countercations on the geometries of NCCL^–^ anions, we reoptimized the geometries in the presence of the 18-crown-6 (18-c-6) complex of the potassium cation (as shown in Scheme S1). Interestingly, the counter cations have little impact on the anion structures (see the italicized values in Figure 1). Therefore, naked anions are studied by employing the M06-2X-D3 method in the subsequent calculations.

**Figure 1:**
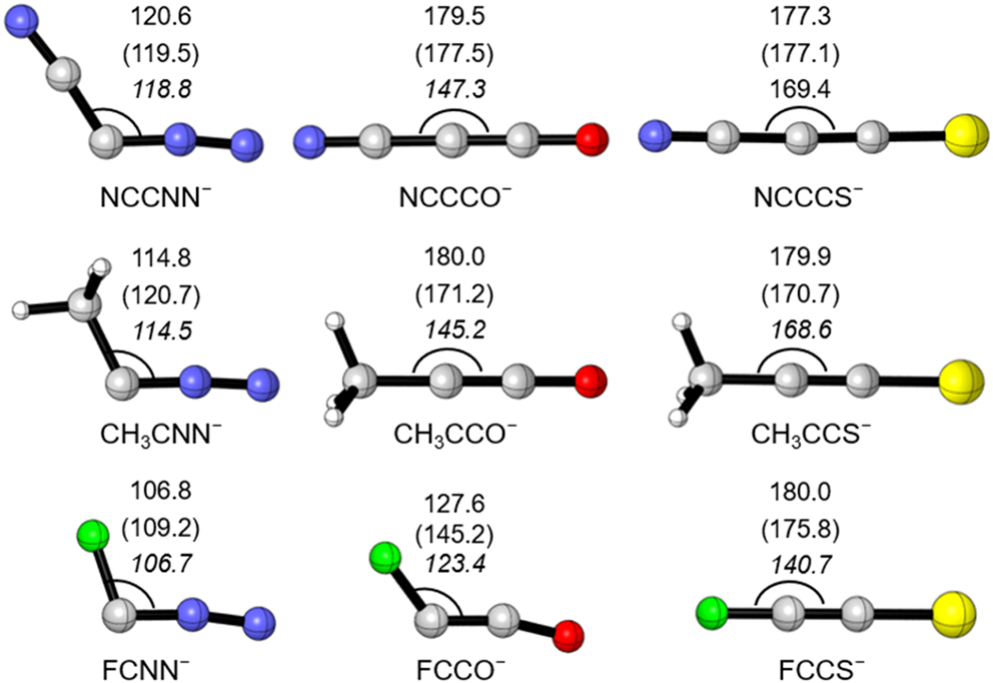
Optimized structures of RCL^–^ (R = NC, CH_3_, and F; L = N_2_, CO, and CS) with bond angles ∠RCL (in degree) listed at the theoretical levels of M06-2X-D3/6-311+G­(d) (normal fonts), the same M06-2X-D3 by incorporating counter cations (in parentheses), and CCSD­(T) (in italics). C, O, N, S, and F are gray, red, blue, yellow, and green, respectively.

The closed-shell singlet state of NCC^–^ involves two degenerate electronic configurations, and the CASPT2/6-311+G­(d) wave function can be expressed as

3 $$\Psi =0.707Â\left[\Psi^{14}{\left(\pi_{//}-1\right)}^{2}{\left(\pi_{⊥}-1\right)}^{2}\pi_{//}^{2}\right]-0.707Â\left[\Psi^{14}{\left(\pi_{//}-1\right)}^{2}{\left(\pi_{⊥}-1\right)}^{2}\pi_{⊥}^{2}\right]$$

where the in-plane π_//_ and out-of-plane π_⊥_ orbitals (shown in Figure 2) are primarily localized on the terminal carbon atom. Visual inspection (see Figure S1) shows that the two highest-lying π_⊥_ and π_⊥_ – 1 orbitals in NCCNN^–^ primarily originate from the NCC^–^ fragment, while the third-highest orbital (denoted as π_⊥_ – 2) localizes predominantly on the N_2_ moiety. Therefore, the NCC^–^ fragment contributes four electrons to the π_⊥_-symmetric orbitals for the NCCNN^–^ anion. Similar electronic configurations exist in the NCCCO^–^ and NCCCS^–^ anions. Consequently, we will employ energy decomposition analysis (EDA) to probe the nature of interactions between the Lewis base L and the closed-shell NCC^–^ anion within the electronic configuration *Â*[Ψ^14^(π_//_ – 1)^2^(π_⊥_ – 1)^2^π_⊥_^2^] featuring four π_⊥_ electrons.

**Figure 2:**
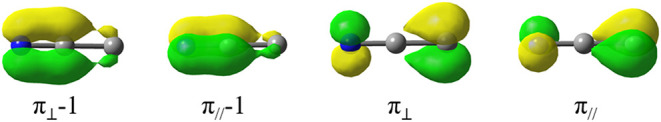
π Molecule orbitals of NCC^–^ at the CASPT2/6-311+G­(d) level.

### Bonding Nature of NCCL^–^ Anions

While various EDA schemes such as EDA-NOCV,^52,53^ GKS-EDA,^54,55^ and SAPT^56^ have been developed and widely applied, here the BLW-ED approach^44,45^ was chosen due to its unique advantage that the charge transfer among interacting moieties can be strictly deactivated. In other words, the σ and π electron transfers between the closed-shell NCC^–^ anion and the L can be quantified individually. Table 1 lists the quantitative data from the BLW-ED analyses.

**Table 1:** Computed Energy Components (kcal/mol) at the M06-2X-D3/6- 311+G­(d) Level with the BLW-ED Approach

complex	Δ*E*_b_	Δ*E*_def_	Δ*E*_int_	Δ*E*_steric_	Δ*E*_pol_	Δ*E*_CT_	Δ*E*_CTπ//_	Δ*E*_CTπ⊥_
NCCNN^–^	–49.3	7.4	–56.7	389.8	–235.1	–211.4	–37.1	–70.6
NCCCO^–^	–111.9	9.4	–120.4	798.6	–678.6	–240.4	–67.3	–67.3
NCCCS^–^	–160.5	7.8	–168.3	791.0	–666.8	–292.5	–80.7	–81.1
CH_3_CNN^–^	–65.8	14.1	–79.9	374.3	–208.2	–246.0	–37.7	–97.7
CH_3_CCO^–^	–123.9	19.4	–143.3	777.5	–644.8	–276.0	–84.1	–84.2
CH_3_CCS^–^	–184.6	17.2	–201.8	773.0	–634.7	–340.1	–103.7	–103.8
FCNN^–^	–46.6	10.9	–57.5	340.0	–158.7	–238.7	–19.3	–119.2
FCCO^–^	–89.2	9.9	–99.1	418.2	–260.0	–259.4	–38.9	–98.3
FCCS^–^	–148.3	14.7	–162.9	709.1	–502.5	–369.5	–114.7	–114.7

The overall binding energies Δ*E*_b_ (−49.3 and −111.9 kcal/mol) for the NCCNN^–^ and NCCCO^–^ anions are close to half of the total (two ligands) bond dissociation energies in C­(NN)_2_ and C­(CO)_2_.^23^ The variation of the binding energies (in absolute values) in NCCL^–^ follows the trend for L as N_2_ < CO < CS. The BLW-ED analyses further show that the steric, polarization, and charge transfer factors are all very significant, although the strong steric repulsion is mostly offset by the stabilizing polarization and charge transfer interactions. Such large numbers are typical for covalent bonds.

Molecular orbital correlation diagrams have been extensively used to elucidate orbital interactions. This kind of traditional correlation diagram is based on the orbital energy changes from isolated monomers to their complex. However, it is expected that orbital energies would change once monomers are put together, even without any orbital (chemical) interactions. This field effect is missing in conventional orbital correlation diagrams but can be accurately quantified by the BLW method. In other words, the BLW method can correlate orbitals of monomers in the existence of other interacting partners and tracks the evolution of orbital energy levels from isolated, deformed, and block-localized monomers in the existence of others to the final complex. We designate the correlations from the block-localized monomers to the complex as “*in situ*” orbital correlations. Figure 3 shows the “*in situ*” orbital correlation diagrams for the cases of NCCL^–^ (L = N_2_, CO, and CS). Computations indicate that the σ orbital, which is largely the lone pair on the terminal C atom, is always doubly occupied at the isolated states (either optimal or deformed) of NCC^–^. When the deformed NCC^–^ and N_2_ are put together, however, there is a remarkable reshuffling of the orbital energy levels for NCC^–^. The σ orbital (largely occupied by the terminal C lone electron pair) is pushed up to become the LUMO, while both in-plane π_//_ and out-of-plane π_⊥_ become doubly occupied. In other words, there is an “orbital swap” for NCC^–^ owing to the steric (Pauli) repulsion from the approaching N_2_, leading to the electronic configuration of NCC^–^ evolving to *Â*[Ψ^12^(π_//_ – 1)^2^(π_⊥_ – 1)^2^π_⊥_^2^π_//_^2^]. Finally, the LUMO of NCC^–^ interacts with the lowest occupied σ orbital of N_2_, which corresponds to the adjacent nitrogen lone pair in the form of NCC^–^ ← L σ-donation, in addition to the π back-donation from NCC^–^ to N_2_. For NCCCO^–^ and NCCCS^–^, the bonding mechanisms remain the same as those for NCCN_2_^–^. Based on the carbon(0) or carbone theory proposed by the Frenking group,^22–25^ the carbon atom in divalent C(0) compounds (or carbones) CL_2_ retains all four valence electrons in two lone-pair orbitals with σ and π symmetries, while the covalent bonds L → C originate from strong donor–acceptor (dative) interactions between Lewis base L and the closed-shell carbon atom. Thus, our block-localized “*in situ*” frontier orbital interaction diagrams (Figure 3) perfectly confirm the carbon(0) or carbone theory.

**Figure 3:**
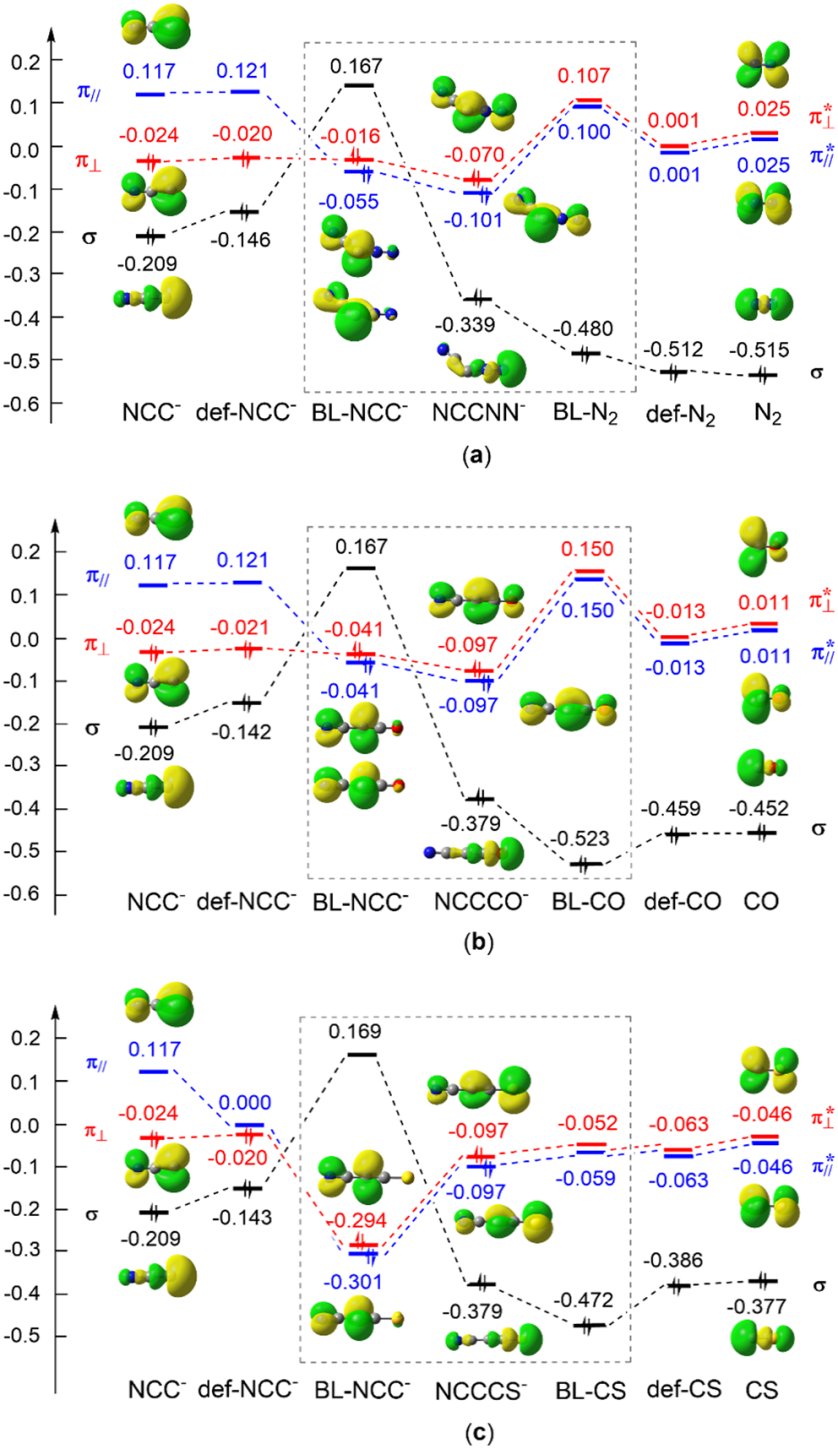
Complete orbital interaction diagrams for (a) NCCNN^–^, (b) NCCCO^–^, and (c) NCCCS^–^, in which def- and BL- refer to deformed and block-localized states with the charge transfer quenched. The gray dashed box corresponds to the *“in situ”* orbital correlation diagram. Energies are in atomic units.

From the perspective of energetics, the electron transfers along the in-plane π_//_ and out-of-plane π_⊥_ pathways stabilize the complex NCCNN^–^ by −37.1 and −70.6 kcal/mol, respectively, and the sum (−107.7 kcal/mol) is close to half of the total charge transfer energy (Δ*E*_CT_ = −211.4 kcal/mol), which includes the NCC^–^ ← L σ-donation. For linear NCCCO^–^ and NCCCS^–^, the π_//_ and π_⊥_ electron transfer energies are nearly equivalent. Consistent with previous studies on CO–transition metal interactions, π back-donation plays a more dominant role in these linear anions.^57,58^ The charge transfer energies of π_//_ (−67.3 and −80.7 kcal/mol, respectively) are significantly larger than that (−37.1 kcal/mol) in the bent NCCNN^–^ anion. Thus, from the perspective of charge transfer energetics in the optimized geometries, our results align with those reported by Gessner et al.^22^ and further indicate that the π-back-donation is stronger for the CS ligand than for the other two systems.

To further elucidate the specific role of the in-plane π_//_ back-donation, we performed BLW geometry optimizations with electron transfer along the in-plane pathways deactivated. Key results are listed in Table 2. Notably, all species adopt very bent geometries. For NCCCO^–^ and NCCCS^–^, their ∠RCL angles reduce to 109.7 and 110.1°, respectively. This contradicts the MO theory predictions of linear structures for these anions, and the data may suggest that π_//_ back-donation exclusively governs the linear geometries in NCCL^–^. However, if we constrain NCCNN^–^ to a linear geometry, the charge transfer energy of π_//_ (−64.5 kcal/mol) would be comparable to the value (−67.3 kcal/mol) in NCCCO^–^. Thus, the present question is: if π_//_ back-donation solely determines the linearity in NCCL^–^ anions, why does NCCNN^–^ deviate from the linear geometry, which exhibits the strongest π_//_ back-donation?

**Table 2:** Optimized Geometries of RCL^–^ with π_//_ Back-Donation Allowed (Y) or Not Allowed (N)

species	π_//_ back-donation	∠RCL	*r*_C–L_	*r*_N–N/C–O/C–S_
N_2_	Y			1.090
CO	Y			1.121
CS	Y			1.529
NCCNN^–^	Y	120.6	1.268	1.148
	N	115.0	1.363	1.135
NCCCO^–^	Y	179.5	1.243	1.202
	N	109.7	1.353	1.167
NCCCS^–^	Y	177.3	1.233	1.634
	N	110.1	1.349	1.581
CH_3_CNN^–^	Y	114.8	1.257	1.170
	N	116.3	1.363	1.153
CH_3_CCO^–^	Y	180.0	1.233	1.231
	N	107.4	1.354	1.186
CH_3_CCS^–^	Y	179.9	1.220	1.679
	N	108.4	1.350	1.604
FCNN^–^	Y	106.8	1.282	1.171
	N	101.4	1.346	1.160
FCCO^–^	Y	127.6	1.279	1.211
	N	102.5	1.369	1.185
FCCS^–^	Y	180.0	1.209	1.688
	N	103.3	1.365	1.594

### Origin of the Significant Difference in the Geometries of NCCL^–^ Anions

To appreciate the correlation of the π_//_ back-donation with the bond angle ∠RCL, we performed constrained DFT optimizations at discrete (fixed) ∠RCL values, followed by BLW computations with the π_//_ back-donation explicitly prohibited. The subsequent DFT and BLW energy profiles along the bond angle ∠RCL are plotted in Figure 4. We note that Figure 4a indicates the BLW energy minimum at ∠RCL ≈ 105°, although the complete BLW optimization leads to a ∠RCL of 115° (Table 2). However, the energy difference between ∠RCL = 115 and 105° is marginal (1.4 kcal/mol). For consistent analyses, we establish the ∠RCL = 115° configuration as the zero-energy reference, where the charge transfer energy of π_//_ back-donation is measured as 34.2 kcal/mol. Enforcing the linearity in NCCNN^–^ results in a significant energy increase of 37.3 kcal/mol at the BLW level, despite the π_//_ charge transfer energy being enhanced to 64.5 kcal/mol. Consequently, the gain of π_//_ electron transfer energy cannot offset the energetic penalty even at the BLW level, rationalizing the bent geometry (∠RCL_2_ = 120.6°) of NCCNN^–^ at the regular DFT level. In contrast, NCCCO^–^ exhibits a 21.1 kcal/mol stabilization for its bent form at the BLW level. Compared with NCCNN^–^, the value is significantly reduced. The increase in the π_//_ electron transfer energy in NCCCO^–^ (from 37.6 to 67.3 kcal/mol) is the same as that of NCCNN^–^. For NCCCS^–^, the enhancement of the π_//_ electron transfer energy becomes more pronounced (from 44.8 to 81.0 kcal/mol), while the linearization penalty (20.2 kcal/mol) aligns with NCCCO^–^. Consequently, the gain of π_//_ electron transfer energy outperforms the energetic penalty at the BLW level, and the linear geometries are preferred by NCCCO^–^ and NCCCS^–^ at the DFT level.

**Figure 4:**
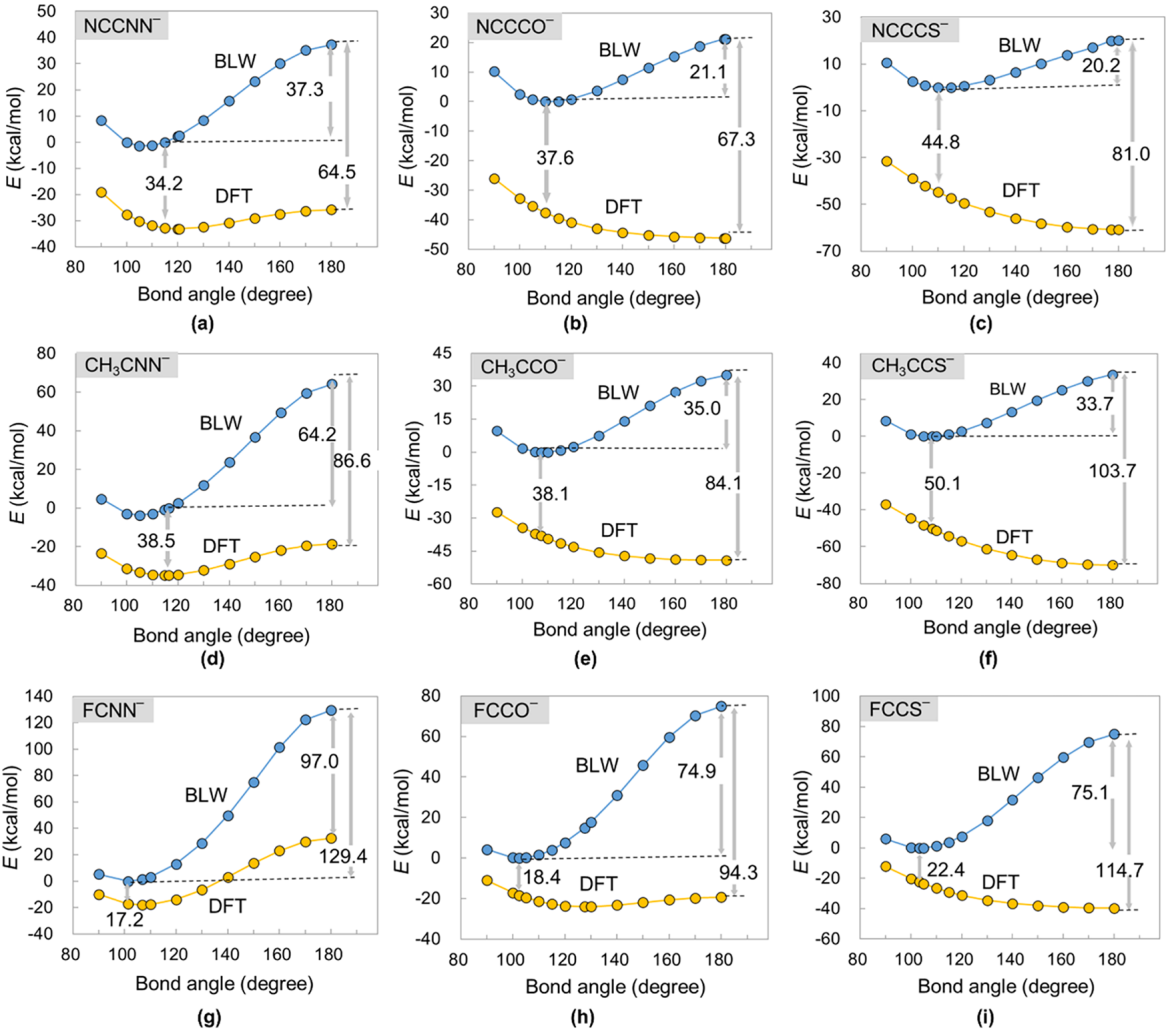
Relative energy profiles with respect to the bond angle ∠RCL (R = NC, CH_3_, and F) when π_//_ back-donation is allowed (DFT) or not (BLW): (a) NCCNN^–^, (b) NCCCO^–^, (c) NCCCS^–^, (d) CH_3_CNN^–^, (e) CH_3_CCO^–^, (f) CH_3_CCS^–^,(g) FCNN^–^, (h) FCCO^–^, and (i) FCCS^–^.

What is the origin of the elevated energy in linear geometries at the BLW level? The BLW-ED analysis (see Table S2) reveals that while the polarization energy (Δ*E*_pol_) and σ and π_⊥_ charge transfer energy (Δ*E*_CTσ+π⊥_) become more favorable upon linearization, the steric energy component (Δ*E*_steric_) increases dramatically. This pronounced rise in Δ*E*_steric_ constitutes the primary destabilizing factor for the linear geometries. Given the above, the geometries of NCCL^–^ (L = N_2_, CO, CS) anions can be explained by the energy balance between the π_//_ electron delocalization and the steric effect.

Figure 5 displays selected in-plane natural localized MOs primarily situated on the central carbon atom or the ligand. For bent NCCNN^–^, the first two orbitals correspond to the σ_C–N_ and σ_C–C_ bonds. The third orbital is a lone pair centered on the central carbon, and the last orbital is an in-plane π_//_ orbital largely delocalized over the N_2_ ligand. The linear NCCCO^–^ and NCCCS^–^ anions exhibit distinct orbital composition. The first two orbitals retain the σ bond around the central carbon, while the third is an in-plane π_//_ orbital primarily localized on the carbon atoms of the NCC^–^ fragment and the CO/CS ligand. The fourth orbital is a lone pair predominantly located on the oxygen or sulfur atom. Even when the ∠RCL angles of NCCCO^–^ and NCCCS^–^ anions are constrained to 120°, a value closely matching the ∠RCL angle in NCCNN^–^, the characteristic orbitals remain notably similar.

**Figure 5:**
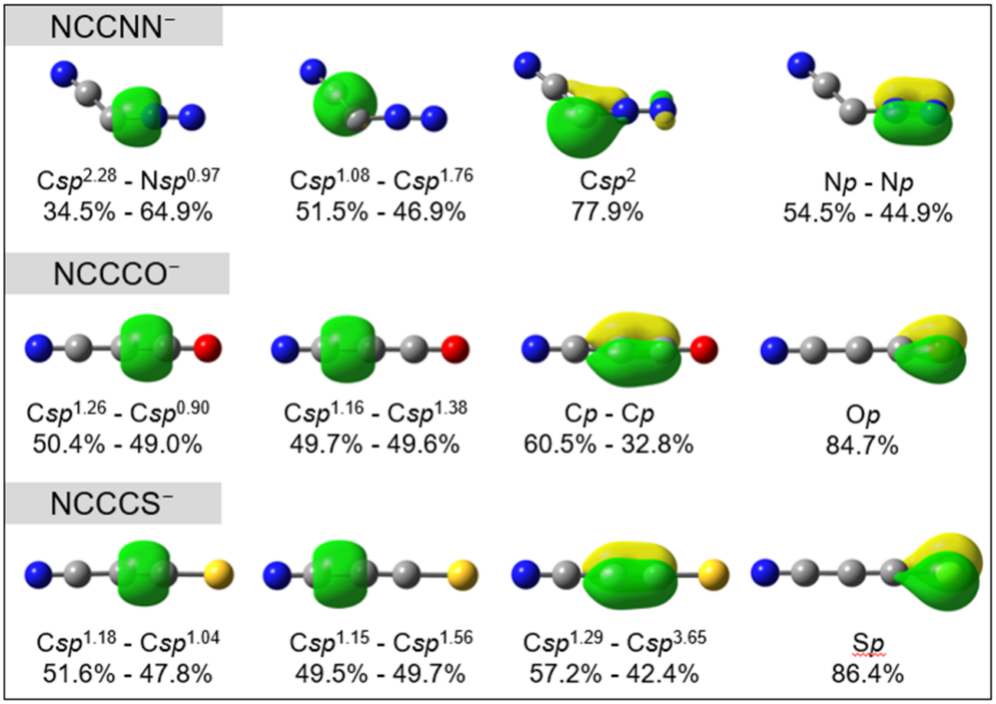
Selected natural localized molecular orbitals with atomic hybridization mode and atomic contributions.

To check the electron repulsion between the π_//_ bond of the ligand (the fourth orbital in Figure 5) and the orbitals around the central carbon atom (the first three orbitals in Figure 5), NSA is employed for NCCL^–^ with respect to the bond angle ∠RCL. For NCCNN^–^, NSA identifies minimal electron repulsion energy at ∠RCL ≈ 115° (Figures 6 and S2), in good agreement with the BLW-optimized geometry (Table 2). Similarly, the electron repulsion favors bent configurations in both NCCCO^–^ and NCCCS^–^, with minimal repulsion energies occurring at 120 and 140°, respectively. These NSA-derived angles diverge slightly from the BLW-optimized geometries due to fundamental methodological differences. NSA employs densities from conventional DFT computations, inherently incorporating π_//_ electron delocalization, which is absent in the BLW treatments. In brief, the electron repulsion between the π_//_ bond of the ligands and the in-plane orbitals around the central carbon atom favors bent geometries, which is consistent with the BLW optimization results.

**Figure 6:**
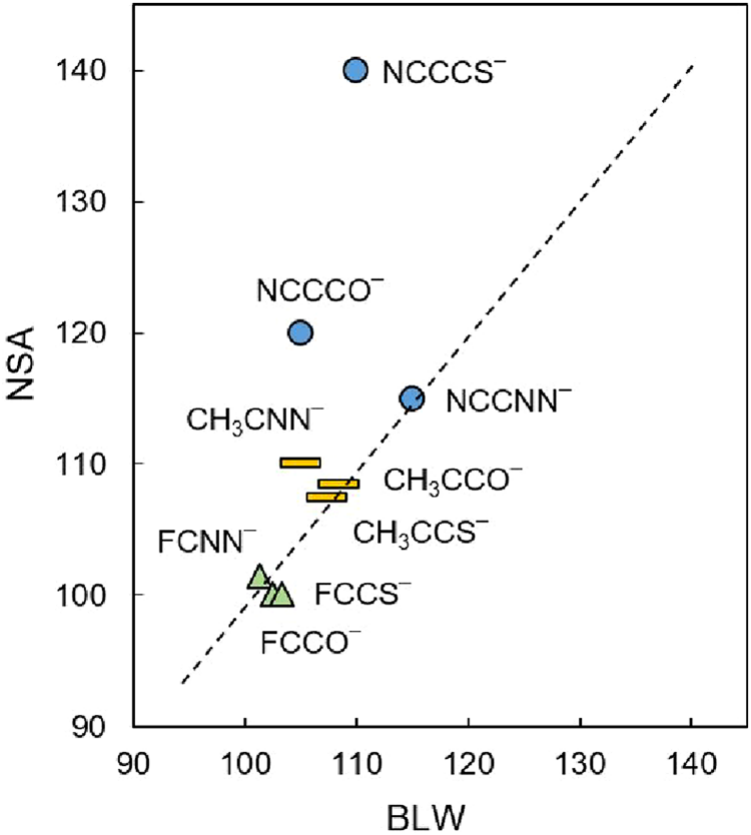
NSA-derived ∠RCL angle with minimum repulsion energy versus BLW-optimized ∠RCL values.

### Substituent Effect on the RCL^–^ Anions

To further explore the substituent effect, we replaced the π-electron-deficient NC group with CH_3_ and F in NCCL^–^ anions. Computational results (Table 2) demonstrate the progressively acute ∠RCL bond angle evolving from NCCNN^–^ (120.6°) to CH_3_CNN^–^ (114.8°) to FCNN^–^ (106.8°), accompanied by substantial increases in the linear-bent energy gap (Figure 4) from 7.3 kcal/mol (NCCNN^–^) to 16.2 kcal/mol (CH_3_CNN^–^) to 50.4 kcal/mol (FCNN^–^). Consequently, the stability for bent geometries is enhanced by the substituent groups CH_3_ and F compared with CN. The linear-bent energy gap expansion at the BLW level coincides with the amplified π_//_ back-donation. This is because, unlike CN, CH_3_ and F groups contain σ_C–H_ electrons or in-plane lone pair that simultaneously intensifies the repulsion between the central carbon orbitals and ligand π_//_ orbital, and the π_//_ back-donation capability. Consistent with NCCNN^–^, the BLW steric penalty increase exceeds the enhancement of the π_//_ charge transfer energy (Figure 4c,f), leading to the bent geometries for CH_3_CNN^–^ and FCNN^–^. NSA-derived ∠RCL angle with minimum repulsion energy is consistent with the BLW-optimized geometry (Figure 6).

For RCCO^–^ anions, the enhancement of the π_//_ back-donation is like the RCNN^–^ systems, while the linear-bent energy gap for the BLW states is less pronounced. For the FCCO^–^ anion, the near-equilibrium between the BLW steric penalty (74.9 kcal/mol) and the π_//_ back-donation energy gain (75.9 kcal/mol) during linearization explains the small (4.6 kcal/mol) bent-linear energy gap observed at the DFT level. For RCCS^–^ anions, the linear-bent energy gaps for the BLW states are comparable to the RCCO^–^ counterparts, but RCCS^–^ displays greater π_//_ back-donation enhancement. Ultimately, both CH_3_CCS^–^ and FCCS^–^ prefer linear geometries.

## Conclusions

In summary, by performing both the regular DFT and BLW computations at the same theoretical level, we demonstrated that the distinct geometries of NCCL^–^ anions (L = N_2_, CO and CS) arise from a competition between π_//_ back-donation and steric repulsion. For the NCCNN^–^ anion, enforcing linearity results in a gain in π_//_ electron transfer stability that is insufficient to make up for the steric penalty at the BLW state, rationalizing its bent geometry at the DFT state. In contrast, for NCCCO^–^ and NCCCS^–^, the steric penalty is reduced, allowing the linear geometries to remain favorable. Consistent with the BLW results, the NSA analyses showed that the electron repulsion between the π_//_ bond of the ligands and the in-plane orbitals around the central carbon atom favors bent geometries. Furthermore, substituting the electron-withdrawing NC group with CH_3_ or F, which contribute in-plane σ-electrons or lone pairs, leads to more acute ∠RCN angles in RCNN^–^, whereas RCCO^–^ and RCCS^–^ retain linear geometries. In the BLW study, we applied the concept of “*in situ*” orbital correlations to the bonding between NCC^–^ and L and identified orbital swaps between an occupied σ orbital corresponding to the lone-pair orbital on the terminal C and an unoccupied in-plane π_//_ orbital due to the steric repulsion with the ligand L. The subsequent “*in situ*” bonding is consistent with the carbon(0) or carbone theory proposed by the Frenking group.^22–25^ We anticipate that the insights gained from this work will facilitate the targeted synthesis and further investigation of the reactivity of the RCL^–^ anions.

## Supplementary Material



## Notes

Comparison of the calculated and experimental structure parameters and the Δ*E*_linear_; the 18-crown-6 (18-c-6) complex of the potassium cation; the π orbitals in NCC^–^ and NCCL^–^ anions; the repulsion energy with respect to the bond angle ∠RCL; and computed energy components (kcal/mol) with the BLW-ED approach (PDF)
