# Supplementary material for: Distinct Difference in the Geometries of NCCL– Anions (L = N2, CO, CS): A Balance Between π Conjugation and Steric Repulsion
Source: Inorg Chem. 2025 Oct 29;64(44):22210–8. doi: 10.1021/acs.inorgchem.5c04439 (PMC12606702; doi:10.1021/acs.inorgchem.5c04439)
Supplement: Supplementary file 1 [file ic5c04439_si_001.pdf]

Supporting Information

The Distinct Difference in the Geometries of $NCCL^-$ Anions (L = N_2, CO, CS): A Balance Between π Conjugation and Steric Repulsion

Jia Wei,[a] Rui Ma,[a] Jinshuai Song,[b] Yandong Duan,[c] Xiaoyan Li[a] Huaiyu Zhang,[*,a] Yirong Mo[*,d]

[a] Institute of Computational Quantum Chemistry, and Hebei Key Laboratory of Inorganic Nanomaterials, College of Chemistry and Materials Science, Hebei Normal University, Shijiazhuang, 050024, China

[b] Green Catalysis Center, and College of Chemistry, Zhengzhou University, Zhengzhou, 450001, China

[c] Hebei Key Laboratory of Photoelectric Control on Surface and Interface, School of Sciences, Hebei University of Science and Technology, Shijiazhuang, 050018, China. [d] Department of Nanoscience, Joint School of Nanoscience and Nanoengineering, University of North Carolina at Greensboro, Greensboro, North Carolina, 27401, United States.

The corresponding author emails: huaiyu.zhang@hebtu.edu.cn, y_mo3@uncg.edu.

Table S1. Comparison of the calculated and experimental structure parameters of RCL^- ($L=N_2$, CO, CS) anions and the ΔE_{linear} (kcal/mol) at CCSD(T) level.

	Structures & Energies	CCSD(T)/6-311+G*	CCSD(T)/6-311+G*-linear	M06-2X-D3/6-311+G(d)	M06-2X-D3/6-311+G(d)[a]	BP86+D3 (BJ)/def2-TZVPP[b]	*exp*[b]
	N1-C2	1.185	1.196	1.167	1.164	1.188	1.51(1)
	C2-C1	1.406	1.339	1.389	1.394	1.373	1.32(2)
**$NCCNN^-$**	C1-N2	1.288	1.233	1.268	1.268	1.269	1.26(2)
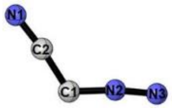	N2-N3	1.172	1.190	1.148	1.150	-	-
	∠C2C1N2	118.8	180.0	120.6	119.5	128.4	133(1)
	E (a.u.)	-239.90176	-239.88296	-	-	-	-
	ΔE_{linear}		11.8	-	-	-	-
	N-C2	1.185	1.187	1.168	1.165	1.189	1.787(3)
	C2-C1	1.373	1.358	1.352	1.356	1.345	1.285(3)
**$NCCCO^-$**	C1-C3	1.271	1.255	1.243	1.241	1.258	1.258(3)
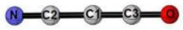	C3-O	1.212	1.218	1.202	1.204	-	-
	∠C2C1C3	147.3	180.0	179.5	177.5	180.0	166.4(2)
	E (a.u.)	-243.77414	-243.77227	-	-	-	-
	ΔE_{linear}		1.2	-	-	-	-
	N-C2	1.184	1.183	1.164	1.163	1.188	-
	C2-C1	1.365	1.363	1.358	1.361	1.346	-
	C1-C3	1.251	1.248	1.233	1.233	1.253	-
**$NCCCS^-$**	C3-S	1.642	1.644	1.634	1.631	-	-
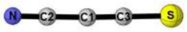	∠C2C1C3	169.4	180.0	177.3	177.1	180.0	-
	E (a.u.)	-566.37324	-566.37255	-	-	-	-
	ΔE_{linear}		0.4	-	-	-	-
	C2-C1	1.527	1.465	1.516	1.508	-	-
**CH_3CNN^-**	C1-N1	1.277	1.224	1.257	1.241	-	-
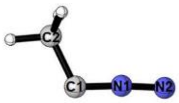	N1-N2	1.193	1.214	1.170	1.180	-	-
	∠C2C1N1	114.5	180.0	114.8	120.7	-	-
	E (a.u.)	-186.99287	-186.95781	-	-	-	-
	ΔE_{linear}		22.0	-	-	-	-
	C2-C1	1.490	1.473	1.463	1.467	-	-
**CH_3CCO^-**	C1-C3	1.266	1.246	1.233	1.224	-	-
	C3-O	1.237	1.248	1.231	1.246	-	-

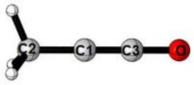	∠C2C1C3	145.2	180.0	180.0	171.2	-	-
	E (a.u.)	-190.85963	-190.85725	-	-	-	-
	ΔE_{linear}		1.5	-	-	-	-
**CH_3CCS^-** 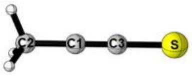	C2-C1	1.473	1.472	1.462	1.464	-	-
	C1-C3	1.239	1.236	1.220	1.222	-	-
	C3-S	1.686	1.689	1.679	1.677	-	-
	∠C2C1C3	168.6	180.0	179.9	170.7	-	-
	E (a.u.)	-513.47519	-513.47445	-	-	-	-
	ΔE_{linear}		0.5	-	-	-	-
**$FCNN^-$** 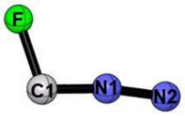	F-C1	1.459	1.361	1.439	1.409	-	-
	C1-N1	1.304	1.215	1.282	1.280	-	-
	N1-N2	1.191	1.222	1.171	1.172	-	-
	∠FC1N1	106.7	180.0	106.8	109.2	-	-
	E (a.u.)	-246.88585	-246.79486	-	-	-	-
	ΔE_{linear}		57.1	-	-	-	-
**$FCCO^-$** 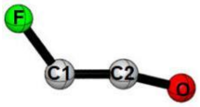	F-C1	1.411	1.352	1.388	1.337	-	-
	C1-C2	1.309	1.232	1.279	1.241	-	-
	C2-O	1.223	1.255	1.211	1.236	-	-
	∠FC1C2	123.4	180.0	127.6	145.2	-	-
	E (a.u.)	-250.73338	-250.71557	-	-	-	-
	ΔE_{linear}		11.2	-	-	-	-
**$FCCS^-$** 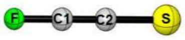	F-C1	1.358	1.329	1.318	1.301	-	-
	C1-C2	1.265	1.225	1.209	1.206	-	-
	C2-S	1.665	1.697	1.688	1.693	-	-
	∠FC1C2	140.7	180.0	180.0	175.8	-	-
	E (a.u.)	-573.34070	-573.33746	-	-	-	-
	ΔE_{linear}		2.0	-	-	-	-

a. The counter cation is included.

b. Data taken from ref [22].

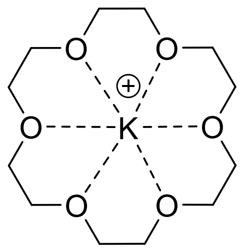

Scheme S1. The 18-crown-6 (18-c-6) complex of the potassium cation.

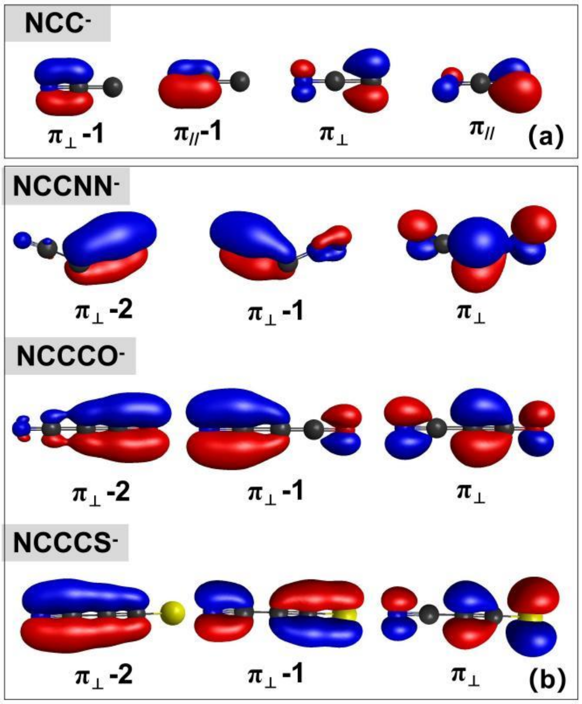

Figure S1. The in-plane $\pi_{//}$ and out-of-plane $\pi_{\perp}$ orbitals in NCC^{-} anion and three highest-lying $\pi_{\perp}$ orbitals in $NCCL^{-}$(L=N_2, CO and CS) anions.

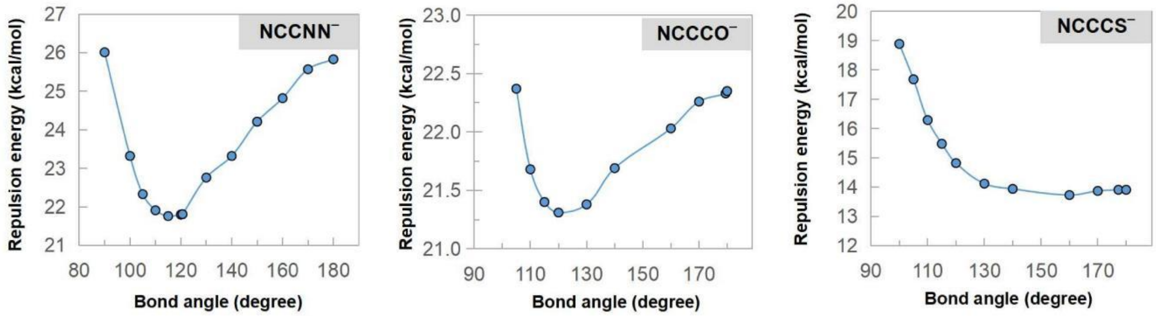

Figure S2. The repulsion energy between the $\pi_{//}$ bond of ligandand the orbitals around central carbon atom with respect to the bond angle∠RCL.

Table S2. Computed energy components (kcal/mol) at the M06-2X-D3/6- 311+G(d) level with the BLW-ED approach.

	Angle	ΔE_b	ΔE_{def}	ΔE_{int}	ΔE_{steric}	ΔE_{pol}	ΔE_{CT}	$\Delta E_{CT\sigma+\pi\perp}$	$\Delta E_{CT\pi//}$
**$NCCNN^-$**	90	-35.1	9.1	-44.2	275.6	-129.4	-190.4	-162.8	-28.7
	100	-43.8	7.9	-51.7	296.0	-152.5	-195.2	-167.4	-28.9
	105	-46.4	7.7	-54.1	312.5	-167.8	-198.8	-169.7	-30.2
	110	-48.1	7.5	-55.6	333.1	-186.0	-202.7	-171.7	-32.0
	115	-49.0	7.4	-56.4	357.5	-207.2	-206.8	-173.7	-34.2
	120	-49.3	7.4	-56.7	385.8	-231.6	-211.0	-175.4	-36.7
	120.63	-49.3	7.4	-56.7	389.8	-235.1	-211.4	-175.5	-37.1
	130	-48.5	7.6	-56.2	453.1	-290.0	-219.3	-178.4	-42.2
	140	-46.8	8.0	-54.8	531.4	-358.9	-227.4	-180.8	-48.1
	150	-44.7	8.6	-53.3	613.8	-432.1	-235.0	-182.8	-53.8
	160	-42.9	9.0	-52.0	688.5	-498.9	-241.5	-184.5	-58.9
	170	-41.7	9.4	-51.1	741.4	-546.2	-246.3	-185.3	-62.8
	180	-41.3	9.5	-50.8	760.3	-563.2	-247.9	-185.3	-64.5
**$NCCCO^-$**	**Angle**	**ΔE_b**	**ΔE_{def}**	**ΔE_{int}**	**ΔE_{steric}**	**ΔE_{pol}**	**ΔE_{CT}**	**$\Delta E_{CT\sigma+\pi\perp}$**	**$\Delta E_{CT\pi//}$**
	90	-91.4	8.6	-100.0	304.9	-190.7	-214.1	-179.1	-36.2
	100	-98.2	7.7	-105.9	315.7	-209.0	-212.6	-178.4	-35.3
	105	-100.8	7.5	-108.4	328.4	-223.2	-213.5	-178.5	-36.1
	110	-103.0	7.5	-110.5	345.6	-241.0	-215.1	-178.6	-37.6
	115	-104.8	7.5	-112.3	367.4	-262.7	-217.0	-178.6	-39.5
	120	-106.2	7.5	-113.8	393.6	-288.5	-218.9	-178.4	-41.7
	130	-108.3	7.8	-116.1	459.1	-352.3	-222.8	-177.4	-46.6
	140	-109.5	8.2	-117.7	539.6	-430.6	-226.6	-176.2	-51.8
	150	-110.2	8.7	-118.9	628.3	-516.5	-230.7	-175.4	-56.7
	160	-110.6	9.1	-119.7	712.9	-597.6	-235.0	-175.5	-61.1
	170	-110.9	9.3	-120.2	775.4	-656.8	-238.8	-175.7	-64.8
	179.48	-110.9	9.4	-120.4	798.6	-678.6	-240.4	-174.8	-67.3
	180	-110.9	9.4	-120.3	798.3	-678.3	-240.4	-174.8	-67.3
**$NCCCS^-$**	**Angle**	**ΔE_b**	**ΔE_{def}**	**ΔE_{int}**	**ΔE_{steric}**	**ΔE_{pol}**	**ΔE_{CT}**	**$\Delta E_{CT\sigma+\pi\perp}$**	**$\Delta E_{CT\pi//}$**
	90	-131.7	8.3	-140.0	328.6	-209.7	-258.9	-218.3	-42.2
	100	-139.1	7.1	-146.2	339.1	-228.2	-257.2	-217.2	-41.4
	105	-142.3	6.8	-149.1	351.7	-242.3	-258.5	-217.2	-42.8
	110	-145.1	6.7	-151.8	368.8	-259.9	-260.6	-217.2	-44.8
	115	-147.6	6.6	-154.2	390.1	-281.2	-263.1	-217.2	-47.4
	120	-149.8	6.6	-156.3	415.6	-306.3	-265.7	-216.9	-50.2
	130	-153.3	7.2	-160.5	477.7	-367.6	-270.7	-215.9	-56.3
	140	-156.0	7.0	-163.0	554.4	-442.0	-275.4	-214.6	-62.5

	150	-157.9	7.3	-165.2	636.9	-521.6	-280.5	-214.0	-68.3
	160	-159.3	7.6	-166.9	714.3	-595.3	-285.9	-214.5	-73.4
	170	-160.1	7.7	-167.9	770.9	-648.2	-290.7	-215.2	-77.6
	177.28	-160.5	7.8	-168.3	791.0	-666.8	-292.5	-214.0	-80.5
	180	-160.5	7.8	-168.3	792.3	-668.0	-292.6	-213.6	-81.0
	**Angle**	**ΔE_b**	**ΔE_{def}**	**ΔE_{int}**	**ΔE_{steric}**	**ΔE_{pol}**	**ΔE_{CT}**	**$\Delta E_{CT\sigma+\pi\perp}$**	**$\Delta E_{CT\pi//}$**
	90	-54.6	14.6	-69.2	296.8	-138.1	-228.0	-197.3	-32.0
	100	-62.4	13.9	-76.2	316.7	-159.6	-233.3	-202.6	-32.0
	105	-64.4	13.8	-78.2	332.5	-173.6	-237.1	-205.2	-33.3
	110	-65.5	13.9	-79.5	352.2	-190.1	-241.6	-207.6	-35.3
	114.8	-65.8	14.1	-79.9	374.3	-208.2	-246.0	-209.7	-37.7
	115	-65.8	14.1	-79.9	375.5	-209.3	-246.2	-209.8	-37.8
**CH_3CNN^-**	116.3	-65.7	14.2	-79.9	390.4	-222.8	-247.4	-210.4	-38.5
	120	-65.4	14.4	-79.8	411.0	-239.7	-251.0	-211.8	-40.8
	130	-63.0	15.1	-78.2	474.1	-291.2	-261.1	-214.9	-47.9
	140	-59.5	16.1	-75.6	546.5	-350.8	-271.2	-216.8	-56.4
	150	-55.5	17.3	-72.7	621.8	-413.5	-281.0	-217.5	-65.7
	160	-51.9	18.4	-70.3	688.0	-468.8	-289.4	-217.0	-74.9
	170	-49.4	19.3	-68.7	733.8	-507.4	-295.0	-214.8	-82.7
	180	-48.5	19.9	-68.4	748.5	-519.9	-297.0	-212.9	-86.6
	**Angle**	**ΔE_b**	**ΔE_{def}**	**ΔE_{int}**	**ΔE_{steric}**	**ΔE_{pol}**	**ΔE_{CT}**	**$\Delta E_{CT\sigma+\pi\perp}$**	**$\Delta E_{CT\pi//}$**
	90	-101.8	14.3	-116.1	742.2	-622.5	-235.7	-201.0	-36.8
	100	-108.8	12.9	-121.6	762.8	-650.0	-234.4	-200.7	-36.0
	105	-111.5	12.8	-124.3	774.6	-663.0	-235.9	-200.9	-37.3
	107.4	-112.5	12.8	-125.4	779.3	-667.9	-236.8	-201.0	-38.1
	110	-113.9	13.0	-126.8	786.2	-674.8	-238.3	-201.2	-39.4
**CH_3CCO^-**	115	-115.9	13.3	-129.2	796.9	-684.8	-241.3	-201.4	-42.2
	120	-117.5	13.8	-131.3	805.7	-692.4	-244.6	-201.5	-45.5
	130	-119.9	15.0	-134.9	815.4	-698.7	-251.6	-201.0	-53.1
	140	-121.4	16.3	-137.7	813.4	-692.7	-258.4	-199.6	-61.3
	150	-122.3	17.5	-139.9	802.9	-678.2	-264.6	-197.9	-69.3
	160	-123.0	18.5	-141.5	790.0	-661.4	-270.1	-196.5	-76.2
	170	-123.6	19.2	-142.7	780.4	-648.8	-274.3	-195.1	-81.5
	180	-123.9	19.4	-143.3	777.5	-644.8	-276.0	-193.9	-84.1
	**Angle**	**ΔE_b**	**ΔE_{def}**	**ΔE_{int}**	**ΔE_{steric}**	**ΔE_{pol}**	**ΔE_{CT}**	**$\Delta E_{CT\sigma+\pi\perp}$**	**$\Delta E_{CT\pi//}$**
	90	-152.1	12.9	-165.1	353.1	-230.5	-287.7	-244.2	-45.4
**CH_3CCS^-**	100	-159.0	11.7	-170.7	429.0	-311.2	-288.5	-245.3	-45.9
	105	-162.4	11.4	-173.8	725.3	-607.7	-291.3	-245.9	-48.2
	108.4	-164.3	11.4	-175.8	768.2	-650.5	-293.5	-246.3	-50.1
	110	-165.5	11.4	-177.0	792.6	-674.5	-295.1	-246.5	-51.5

	115	-168.4	11.6	-180.0	801.0	-681.6	-299.5	-247.1	-55.3
	120	-171.0	11.9	-183.0	807.6	-686.6	-303.9	-247.4	-59.5
	130	-175.4	12.9	-188.3	813.5	-689.1	-312.6	-247.0	-68.6
	140	-178.7	14.0	-192.8	809.5	-681.7	-320.5	-245.5	-77.9
	150	-181.2	15.2	-196.4	798.8	-667.7	-327.5	-243.8	-86.5
	160	-183.0	16.1	-199.1	786.5	-651.9	-333.7	-242.5	-94.0
	170	-184.1	16.8	-201.0	777.0	-639.7	-338.3	-241.2	-99.9
	180	-184.6	17.2	-201.8	773.0	-634.7	-340.1	-238.9	-103.7
**$FCNN^-$**	**Angle**	**ΔE_b**	**ΔE_{def}**	**ΔE_{int}**	**ΔE_{steric}**	**ΔE_{pol}**	**ΔE_{CT}**	**$\Delta E_{CT\sigma+\pi\perp}$**	**$\Delta E_{CT\pi//}$**
	90	-38.7	12.3	-51.0	295.4	-130.2	-216.2	-202.9	-15.3
	100	-46.0	10.9	-56.9	318.3	-145.2	-229.9	-214.6	-16.7
	101.4	-46.4	10.9	-57.3	322.4	-147.8	-231.9	-216.1	-17.2
	105	-46.5	10.9	-57.4	333.7	-154.9	-236.3	-219.8	-18.5
	106.8	-46.6	10.9	-57.5	339.9	-158.7	-238.7	-221.5	-19.3
	110	-46.3	11.1	-57.5	351.8	-166.0	-243.2	-224.4	-20.8
	120	-42.6	11.9	-54.5	395.2	-192.9	-256.8	-231.8	-27.0
	130	-35.2	12.9	-48.1	448.2	-225.9	-270.4	-237.0	-35.4
	140	-25.5	14.0	-39.5	508.7	-263.5	-284.6	-240.1	-46.7
	150	-14.9	15.4	-30.2	571.9	-302.2	-299.9	-240.8	-61.3
	160	-5.1	16.6	-21.8	627.8	-335.1	-314.5	-238.6	-78.2
	170	1.8	17.5	-15.7	665.4	-357.5	-323.6	-233.8	-92.2
	180	4.3	17.7	-13.5	677.7	-365.2	-326.0	-231.5	-96.9
**$FCCO^-$**	**Angle**	**ΔE_b**	**ΔE_{def}**	**ΔE_{int}**	**ΔE_{steric}**	**ΔE_{pol}**	**ΔE_{CT}**	**$\Delta E_{CT\sigma+\pi\perp}$**	**$\Delta E_{CT\pi//}$**
	90	-76.4	10.5	-86.9	291.1	-161.4	-216.6	-203.4	-15.2
	100	-83.3	8.4	-91.7	306.9	-172.7	-225.9	-209.9	-17.3
	102.5	-84.5	8.2	-92.7	312.7	-176.8	-228.6	-211.6	-18.4
	105	-84.7	8.1	-92.9	319.4	-181.6	-230.7	-213.2	-19.7
	110	-86.6	8.1	-94.7	335.1	-193.0	-236.8	-216.1	-22.9
	115	-88.0	8.4	-96.3	354.3	-207.4	-243.2	-218.7	-26.7
	120	-89.5	8.9	-98.4	377.0	-225.1	-250.4	-220.7	-31.1
	127.6	-89.2	9.9	-99.1	418.2	-257.9	-259.4	-222.7	-38.9
	130	-89.8	10.2	-100.0	432.7	-269.7	-263.0	-223.0	-41.7
	140	-88.8	12.0	-100.8	499.4	-324.9	-275.3	-223.0	-54.2
	150	-86.9	13.9	-100.8	570.0	-384.7	-286.1	-220.9	-67.6
	160	-85.5	15.5	-101.0	634.3	-440.1	-295.2	-217.5	-80.3
	170	-84.5	16.6	-101.1	679.7	-479.7	-301.1	-213.8	-90.0
	180	-84.1	17.0	-101.2	695.8	-493.8	-303.2	-211.6	-94.3
**$FCCS^-$**	**Angle**	**ΔE_b**	**ΔE_{def}**	**ΔE_{int}**	**ΔE_{steric}**	**ΔE_{pol}**	**ΔE_{CT}**	**$\Delta E_{CT\sigma+\pi\perp}$**	**$\Delta E_{CT\pi//}$**
	90	-121.5	7.4	-128.8	312.7	-176.8	-264.7	-249.2	-18.0
	100	-129.3	5.2	-134.5	330.2	-190.6	-274.1	-256.3	-20.4

	103.3	-131.6	4.9	-136.6	339.1	-197.2	-278.4	-258.7	-22.4
	105	-132.7	4.9	-137.6	344.2	-201.0	-280.7	-259.8	-23.6
	110	-136.5	5.0	-141.5	361.5	-214.1	-288.9	-263.0	-27.8
	115	-139.0	5.3	-144.3	382.2	-230.1	-296.5	-265.5	-32.9
	120	-141.1	5.9	-147.0	406.2	-248.9	-304.3	-267.5	-38.8
	130	-143.5	7.6	-151.0	462.8	-294.5	-319.4	-269.6	-52.7
	140	-146.0	9.6	-155.5	527.6	-347.7	-335.4	-269.5	-68.5
	150	-146.8	11.6	-158.3	593.9	-403.3	-348.9	-267.6	-84.5
	160	-147.7	13.2	-161.0	652.7	-453.5	-360.1	-264.6	-98.6
	170	-148.1	14.3	-162.3	694.0	-489.3	-367.1	-261.1	-109.2
	180	-148.3	14.7	-162.9	709.1	-502.5	-369.5	-258.1	-114.7
